# Editorial: The “Connectivome Theory”: psyche, soma and the systemic involvement of connective tissue in neurodivergence

**DOI:** 10.3389/fpsyt.2024.1436796

**Published:** 2024-06-20

**Authors:** Leonardo Zoccante, Marco Zaffanello, Gianfranco Di Gennaro

**Affiliations:** ^1^ Azienda Ulss 9 Scaligera, Verona, Italy; ^2^ Department of Surgery, Dentistry, Paediatrics and Gynaecology, University of Verona, Verona, Italy; ^3^ Science of Health Department, School of Medicine, Magna Graecia University of Catanzaro, Catanzaro, Italy

**Keywords:** autism spectrum disorder, connective tissue, psyche, soma, neurodevelopment

The Research Topic *“The ‘Connectivome Theory’: Psyche, Soma and the Systemic Involvement of Connective Tissue in Neurodivergence”* explores the interactions between the different body systems according to the interpretative model of the “Connectivome Theory” (Zoccante et al.), identifying the connective tissue as the common element that could provide an explanation regarding the coexistence of functional alterations in multiple organs in neurodivergences and particularly in autism spectrum disorder. The connective tissue, in its dense, loose and fluid components, constitutes the structural configuration of the body but also makes the mutual influence of one system on another possible. The role of the connective tissue, in fact, is not only that of support but also connection and regulation of the body systems. In autism spectrum disorder, which until a few years ago was reported as a pervasive developmental disorder, there is a parallel “pervasive” involvement of multiple organs. On a clinical level, the element that characterizes the manifestation is the variability of the symptoms. The Research Topic, with the six published contributions, attempts to highlight how the dysfunctional vision of multiple systems contributes to supporting the pervasiveness of autism spectrum disorder.

In the article “Intestinal metabolites and The Risk of Autistic Spectrum Disorder: A Two-sample Mendelian Randomization Study” the authors (Liu et al.) explore the potential causal relationship between 10 intestinal flora-dependent metabolites and autism, finding a potential causality on ASD in two of them (serotonin, which seems to increase the risk of ASD, and choline, which on the contrary could reduce the risk of ASD).

Yano and Hosokawa discuss the relationship that exercise, nutrition and sleep have with the immune, musculoskeletal and gut systems according to the “Connectivome Theory” in their mini review “The Importance of Comprehensive Support Based on the Three Pillars of Exercise, Nutrition, and Sleep for the Improve Core Symptoms of Autism Spectrum Disorders”.

In the exploratory study “Visual-motor involvement in Autism Spectrum Disorder: could the stereopsis deficit affect motor coordination?” (Longo et al.) the visual-motor status was evaluated in a group of 253 children, 203 of which had a diagnosis of ASD, finding that convergence insufficiency and refractive errors are the most observed ocular conditions in autism, which is consistent with the known alterations of motor skills and sensory processing found in ASD.

The article “National survey on bladder and bowel dysfunctions in autism spectrum disorder population” by Gubbiotti et al. reports the results of a national survey aimed at investigating lower urinary tract symptoms (LUTS) and bowel disorders in a population of young subjects with autism spectrum disorder, finding that the prevalence of urinary symptoms is related to higher severity of the ASD condition.

In the paper “Stretch Marks: A Visible Expression of Connective’s Involvement in Autism Spectrum Disorders” the authors Veronese et al. hypothesize what other factors in addition to obesity can determine the phenomenon of stretch marks in people with ASD and what impact this has on the symptomatic picture of autism in terms of behavior, posture and motor skills.

Lastly, the article “Behavioral guidance for improving dental care in autistic spectrum disorders” by Pastore et al. addresses the topic of oral health in children with ASD who, due to behavioral manifestations, represent a challenge for dentists and hygienists, evaluating the long-term effectiveness of behavioral supports and finding a significant improvement in their compliance to regular dentistry visits and treatments.

From a comprehensive reading of the six articles presented in this Research Topic, the central role that connective tissue (and by extension its alterations) has in autism spectrum disorders emerges unequivocally; from this starting point, future studies could further expand our knowledge on the relationship between ASD and alterations in connectivity.

## Author contributions

LZ: Conceptualization, Project administration, Supervision, Writing – original draft, Writing – review & editing. MZ: Validation, Visualization, Writing – review & editing. GD: Supervision, Validation, Writing – review & editing.

